# Left atrial appendage closure: outcomes and challenges

**DOI:** 10.1007/s12471-016-0929-0

**Published:** 2016-12-09

**Authors:** H. S. Suradi, Z. M. Hijazi

**Affiliations:** 1 St Mary Medical Center, Community HealthCare Network, Hobart, IN USA; 2 Community Hospital, Community HealthCare Network, Munster, IN USA; 3 Rush Center for Structural Heart Disease, Rush University Medical Center, Chicago, IL USA; 4 Sidra Cardiac Program, Sidra Medical & Research Center, Doha, Qatar

**Keywords:** Atrial fibrillation, Stroke, Device closure

## Abstract

Whereas the left atrial appendage plays a rather minor role under physiological circumstances, it gains an importance in patients with atrial fibrillation. Compelling evidence has revealed that the left atrial appendage is implicated as the source of thrombus in the vast majority of strokes in atrial fibrillation. Oral anticoagulation remains the standard of care for stroke prevention in atrial fibrillation; nevertheless, this treatment has several limitations and is often contraindicated, particularly in the elderly population in whom the risk of stroke is high. Therefore, occluding the left atrial appendage is a logical approach to prevent thrombus formation and subsequent cardioembolic events in these patients. We present a review of clinical outcomes of patients with atrial fibrillation undergoing left atrial appendage closure and the challenges faced in this field.

## Introduction

Atrial fibrillation (AF) is the most common clinically relevant cardiac arrhythmia with an overall incidence of 0.4–1% in the general population, which tends to increase with age [1]. In 2010, the estimated global prevalence of AF was 33.5 million with a 25% lifetime risk of developing AF among people 40 years of age and older. This prevalence is projected to at least double by 2050 due to the ageing population, presenting significant challenges for health care delivery [2].

Whether paroxysmal, persistent or permanent, AF carries a five-fold increase in the incidence of embolic stroke and a three-fold risk of mortality [3]. AF is responsible for approximately 25% of all ischaemic strokes, occurring in 5% of non-anticoagulated patients every year [4]. Compared with non-AF-related strokes, cardioembolic strokes due to AF tend to result in worse outcomes with the associated mortality rate up to 30% at 12 months and a greater risk of permanent neurological disability [5].

Anticoagulation therapy has been and remains the mainstay for stroke prevention in AF. Nevertheless, a significant number of patients have relative or absolute contraindications to anticoagulation therapy. Compelling evidence has revealed that the left atrial appendage (LAA) is implicated as the source of thrombus in more than 90% of strokes in AF [6]. Therefore, patients with contraindications for anticoagulation therapy may benefit from LAA exclusion.

Recently, there has been significant progress in the innovation of non-surgical methods for the occlusion of the LAA with the hope of reducing strokes. This article aims to provide an update on the clinical outcomes of the most frequently studied devices for left atrial appendage closure and discuss the challenges and future directions of this field.

## Rationale for LAA closure

Based on several randomised clinical trials, anticoagulation has been the cornerstone of stroke prevention in patients with AF who have an elevated stroke risk. The individualised assessment of the risk-benefit balance is central to decision making around pharmacotherapy for stroke reduction in AF. To estimate stroke risk and subsequent need for possible anticoagulation, all major guidelines recommend utilisation of the CHA2DS2-VASc score (Congestive heart failure, Hypertension, Age ≥75 years [doubled], Diabetes mellitus, prior Stroke or TIA or thromboembolism [doubled], Vascular disease, age 65 to 74 years, sex category), which provides an estimate of the potential benefits of therapy [3]. The 2014 American Heart Association/American College of Cardiology/Heart Rhythm Society guideline recommends oral anticoagulation (OAC) therapy for CHA2DS2-VASc score of 2 or greater (estimated annual stroke risk of 2.2%). Patients with a score of 1 could be treated with either OAC, antiplatelet therapy, or receive no treatment [7]. Similarly the recently published 2016 European Society of Cardiology guidelines for the management of AF recommend that OAC should be started in all patients with a CHA2DS2-VASc score of 2 or greater. In patients with a CHA2DS2-VASc score of 1, OAC could be considered after assessing the bleeding risk [8].

Although oral anticoagulation remains the gold standard approach for prevention of thromboembolic stroke in patients with AF, this treatment is often underused for various clinical reasons. Warfarin has a narrow therapeutic window, requires frequent monitoring, has significant drug and food interactions, increases the risk of bleeding and has a high discontinuation rate. Novel oral anticoagulants (NOACs) have been shown to be non-inferior or superior to warfarin but are also associated with increased bleeding risk and costs as well as high discontinuation rates ranging between 17–25% at 2 years [9–11]. Furthermore, AF patients with concomitant coronary artery stents present significant challenges as the addition of oral anticoagulant to dual antiplatelet therapy increases the risk of bleeding and the use of antiplatelet therapy alone does not provide effective stroke prevention [12]. This large unmet clinical need for stroke reduction establishes the foundation for non-pharmacological strategies to prevent embolism in AF. Because most intracardiac thrombus occurs in the LAA in patients with non-valvular AF, a concept of occlusion of the LAA has emerged as an alternative option for stroke prevention [6]. This theory has led to the development of a variety of LAA closure techniques as possible alternative means in patients who are ineligible for anticoagulation.

**Fig. 1:**
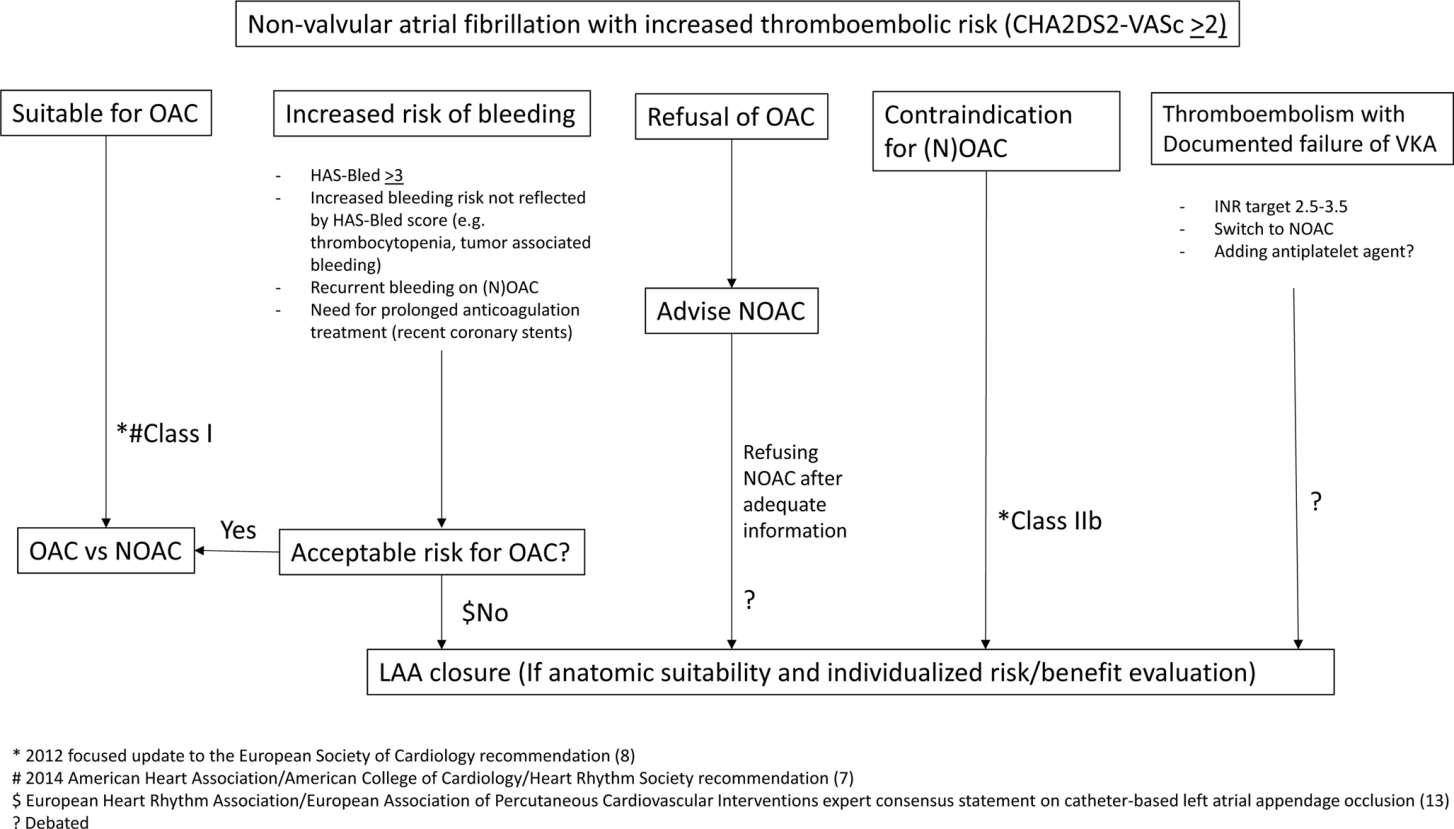
Algorithm of stroke prevention in atrial fibrillation. *LAA* left atrial appendage; *NOAC* novel oral anticoagulant; *OAC* oral anticoagulant; *VKA* vitamin K antagonist

Guideline recommendations for LAA occlusion for stroke prevention are limited due to the lack of data from clinical trials for any of these devices aside from the WATCHMAN. The current ACC/AHA/HRS Guideline for the Management of Patients with Atrial Fibrillation does not specify the indications for catheter-based LAA exclusion techniques [7]. The 2016 European Society of Cardiology Guidelines for the Management of Atrial Fibrillation [8] gives a Class IIb recommendation (usefulness/efficacy is less well established by evidence/opinion) for percutaneous LAA closure in patients who are at high stroke risk and have contraindications for long-term oral anticoagulation [8]. The European Heart Rhythm Association/European Association of Percutaneous Cardiovascular Interventions issued a consensus statement on catheter-based LAA occlusion in 2014 stating that LAA exclusion had the clearest indication in patients who had an absolute contraindication to anticoagulation and possible indication in those with an increased risk of bleeding [13]. These recommendations may evolve with subsequent revisions given the developments in LAA occlusion since the time the existing guidelines were written. Fig. 1 illustrates clinical pathways for stroke prevention in non-valvular AF.

## LAA closure for stroke reduction

### Surgical approach for LAA closure

Surgical exclusion of the LAA is typically reserved for patients undergoing cardiac surgery for valvular and coronary artery disease. LAA exclusion is often performed in patients without AF and is combined with valvular surgery despite the fact that thromboembolism in valvular AF is related to the LAA in only 60% of cases, making it less ideal for LAA closure. Techniques vary from appendectomy to staple or suture exclusion with significant variability in completeness of LAA closure, reported to be between 0 and 100% [14, 15]. Because of the wide variability in surgical methods, completeness of LAA closure and the heterogeneity of patients with AF, the effectiveness of adjunctive surgical exclusion of LAA is not well established. Current data on the effectiveness of surgical excision on reducing stroke in patients with AF is limited to observational and retrospective studies. The ACC/AHA/HRS and ESC Atrial Fibrillation Guidelines both give a Class IIb recommendation for surgical LAA closure [7]. The Left Atrial Appendage Occlusion Study III (LAAOS III) is an ongoing randomised controlled trial to evaluate LAA occlusion during cardiac surgical procedures (clinicaltrials.gov NCT01561651). The endpoint will be first occurrence of stroke or systemic arterial embolism over a mean follow-up of 4 years. Surgical techniques to occlude the LAA continue to evolve, with efforts being made to overcome the inconsistent closure and intrathoracic bleeding associated with these techniques. Surgical obliteration of the LAA is typically not performed as a stand-alone procedure, raising the need for a minimally invasive percutaneous approach.

### Transcatheter approaches for LAA closure

**Fig. 2:**
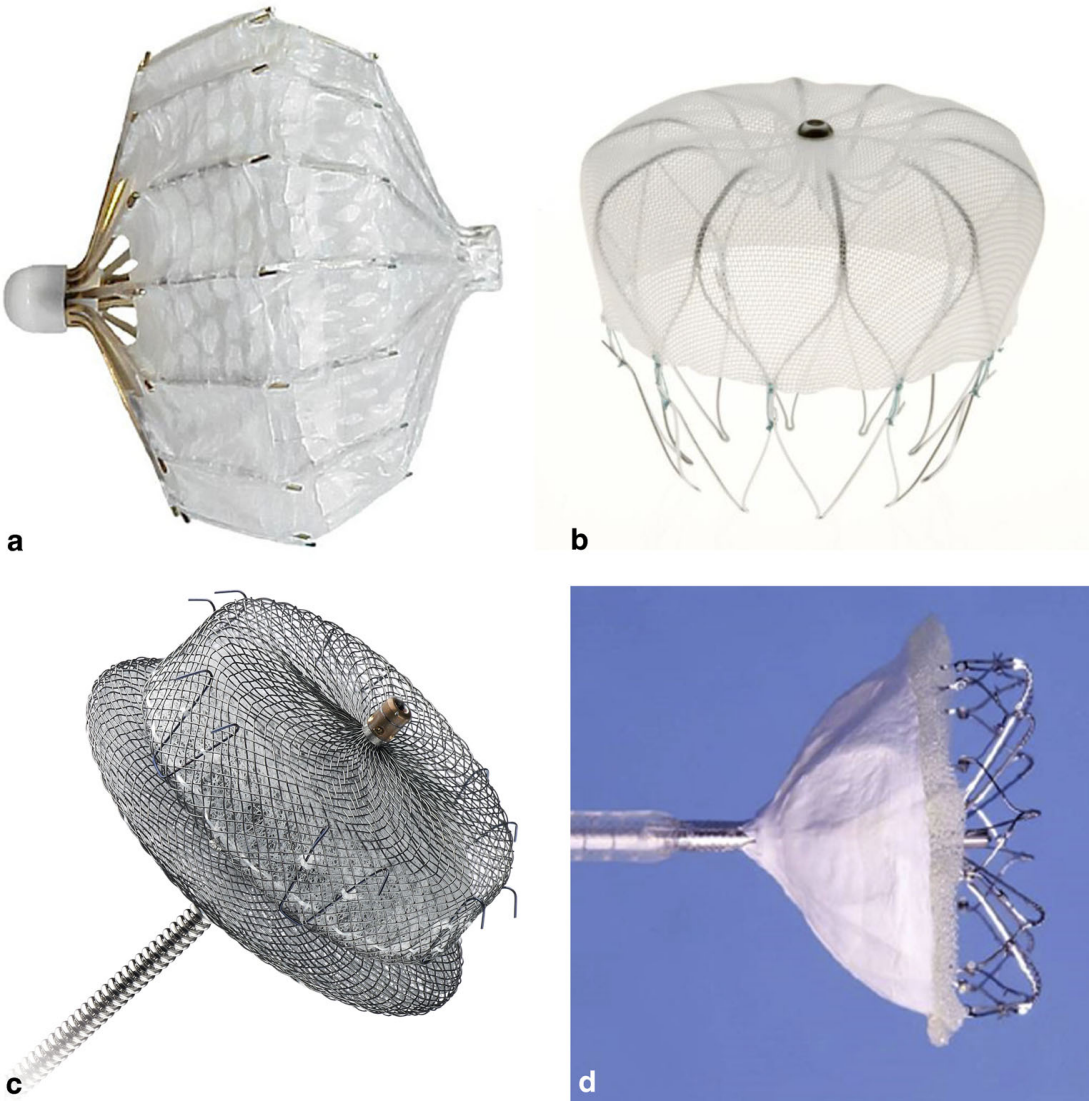
Endovascular left atrial appendage closure devices. **a** PLAATO (Courtesy of Boston Scientific), **b** WATCHMAN (Courtesy of Boston Scientific), **c** Amplatzer Cardiac Plug (Courtesy of St. Jude Medical), **d** Wavecrest (Courtesy of Coherex Medical)

Percutaneous LAA closure was introduced into clinical practice in 2001 using the PLAATO system (Percutaneous Left Atrial Appendage Transcatheter Occlusion; Appriva Medical INC, Sunnyvale, California, USA) [16]. The implant comprised a self-expanding nitinol cage with an occlusive expanded polytetrafluoroethylene membrane directly laminated to the frame structure to exclude the appendage from the circulation (Fig. 2a). The PLAATO device had a number of significant drawbacks and the implantation technique was fairly difficult and perilous. The device was later withdrawn from the market in 2006. However, the concept of percutaneous closure of the appendage was proved.

Currently, there are multiple percutaneous approaches which can be classified into two broad categories: endocardial plug devices to occlude the ostium of the LAA and epicardial LAA ligation procedures to exclude the LAA (Table 1). The WATCHMAN is currently the only percutaneous occlusion device approved in the United States. Several devices including the Amplatzer, WATCHMAN, Wavecrest, LAmbre, Occlutech and LARIAT devices have the Conformité Européenne (CE) mark of approval. Below we discuss the clinical outcomes of the most frequently studied devices.

**Table 1:** Currently available left atrial appendage closure devices

Device (Manufacturer)	Design	Device Sizes (mm)	Sheath Size	Approval Status
*Endocardial devices*				
PLAATO (Appriva Medical)	Single-lobe occluder; nitinol cage; ePTFE membrane; hooks	15, 18, 20, 23, 26, 29, and 32 mm	14 French	Removed from market
Watchman (Boston Scientific)	Single-lobe occluder with nitinol frame, ePTFE membrane, 10 hooks	21, 24, 27, 30, 33	14 French	CE Mark (2005) FDA approval (2015)
Amplatzer Cardiac Plug (St. Jude Medical)	Lobe & disc; nitinol mesh construct; Stabilising wires	16, 18, 20, 22, 24, 26, 28, 30	9, 10, 13 French	CE Mark (2008)
Amulet (St Jude Medical)	Wider lobe and disc, more stabilising wires compared to ACP	16, 18, 20, 22, 25, 28, 31, 34	14 French	CE Mark (2013)
WaveCrest (Coherex Medical)	Single-lobe occluder. Nitinol frame, polyurethane foam, ePTFE membrane, retractable anchors	22, 27, 32	–	CE Mark (2013)
Sideris Transcatheter Patch (Custom Medical Devices)	Frameless detachable latex balloon covered with polyurethane	–	–	Not yet
Occlutech LAA Occluder (Occlutech)	Single-lobe occluder. Nitinol wire mesh, stabilizing loops	15, 18, 21, 24, 27, 30, 33, 36, 39	12, 14 French	CE Mark (2016)
Lambre (Lifetech)	Lobe and disc nitinol frame. PET membrane. Distal barbs anchors	16 to 36	7–10 French	CE Mark (2016)
Cardia Ultrasept (Cardia)	Lobe and disc nitinol frame. Ivalon covering. Distal anchors	16, 20, 24, 28, 32	10, 11 and 12 French	Not yet
*Epicardial Devices*				
Lariat (SentreHeart)	Endocardial and epicardial percutaneous access required	LAA sizes up to 40 width, 20 height, 70 length	14 French	CE Mark (2015) FDA approval (for tissue apposition)
AEGIS (AEGIS Medical Innovations)	Epicardial approach. Electrode-guided navigation for LAA tissue capture	–	–	Not yet

*ePTFE* expanded polytetrafluoroethylene, *LAA* left atrial appendage, *PET* polyethylene terephthalate

## Endocardial LAA closure devices

### WATCHMAN

The WATCHMAN device (Boston Scientific, Natick, MA, USA) is a self-expanding nitinol device composed of a polyethylene terephthalate (PTFE) membrane on its proximal surface that filters blood entering and leaving the appendage. Fixation barbs surround the mid-portion of the device to engage the LAA wall (Fig. 2b). The WATCHMAN device is the only device that has undergone rigorous scientific evaluation and has received both CE and the US Food and Drug Administration (FDA) approval. The WATCHMAN device has been evaluated in two randomised clinical trials: the PROTECT-AF (WATCHMAN Left Atrial Appendage System for Embolic Protection in Patients with Atrial Fibrillation) and PREVAIL (Prospective Randomized Evaluation of the WATCHMAN Left Atrial Appendage Closure Device in Patients with Atrial Fibrillation vs long-term Warfarin therapy) trial [17].

PROTECT AF trial randomised 707 patients with non-valvular atrial fibrillation in a 2:1 ratio to either the device or long-term warfarin therapy with a primary combined endpoint of all-stroke, systemic thromboembolism and cardiovascular death. Patients randomised to the device arm were placed on warfarin and aspirin for 45 days post implantation and then underwent repeat transoesophageal echocardiogram (TEE). Warfarin was discontinued in those patients who either had complete closure of the LAA or a small peri-device leak (jet <5 mm in width). After discontinuation of warfarin, dual antiplatelet therapy with aspirin and plavix was continued until the 6‑month follow-up, followed by aspirin alone indefinitely.

After a mean follow-up of 2.3 years, the primary efficacy event rates were similar in the device and warfarin therapy groups (3.0 and 4.3%, respectively) demonstrating non-inferiority of the device compared with warfarin therapy [17]. At a mean follow-up of 3.8 years, the primary efficacy rate remained similar in both groups (2.3% vs. 3.8%, respectively) with the device meeting superiority with regard to all-cause mortality (3.2% vs. 4.8%) and cardiovascular mortality (1% vs. 2.4%, respectively) which was predominantly driven by a significant reduction in haemorrhagic stroke (0.2% vs. 1%, respectively) [18]. The device was successfully implanted in 88% of patients randomised to the WATCHMAN group and in 91% of patients in whom implant was attempted. At the 45-day and 6‑month follow-up TEE, 86% and 92% of patients were able to discontinue warfarin, respectively. In a retrospective review of 6‑ and 12-month follow-up echocardiograms in over 400 patients who received the WATCHMAN device, there was no significant increase in the rate of thromboembolism in the 32% of patients with residual peri-device leak compared with those without [19], suggesting that most residual holes are small and not associated with embolisation of large clots.

PROTECT AF has demonstrated efficacy and confirmed long-term safety; however, acute safety events were a concern. The primary safety endpoint at 2 years occurred significantly more often in the device group (10.2% vs. 6.8%, respectively) with the most frequent primary complications being pericardial effusion and ischaemic stroke. To obtain further efficacy and particularly safety data, the WATCHMAN device was studied in the Continuous Access Protocol (CAP) registry by experienced operators who participated in PROTECT AF trial [20]. The results of the CAP registry confirmed a higher procedural success rate (95% vs. 88%) and significant decline in the rate of safety events compared with the PROTECT AF trial (3.7% vs. 7.7%; *p* = 0.007). Importantly, there were no periprocedural strokes, and the pericardial effusion rate was only 2.2% compared with 5.5% in the PROTECT AF trial. This suggested the possibility of improved outcomes with device implantation with increased operator experience.

The PREVAIL study was designed similarly to strengthen the results of the PROTECT AF trial in patients at somewhat higher risk treated by centres with variable experience. This study randomly assigned 407 patients in a 2:1 ratio to WATCHMAN or warfarin. Antithrombotic regimen post-implantation was similar to PROTECT AF. The WATCHMAN device was successfully implanted in 95.1% of patients in whom implant was attempted, an improvement from PROTECT AF (*p* = 0.04). Furthermore, 39.1% of implants were performed by new operators with no statistically significant difference in success or complications compared with experienced operators, demonstrating improvements in physician education and the evolution of the procedure. All 7‑day complications after attempted implantation occurred at a significantly lower rate in PREVAIL compared with PROTECT AF (4.5% vs. 8.7%, *p* = 0.004). These data were consistent with data from the CAP registry demonstrating procedural complications as infrequent and significantly improved.

At 18-month follow-up, the first co-primary efficacy endpoint (composite of stroke, systemic embolism, and cardiovascular/unexplained death) was 0.064 with WATCHMAN versus 0.063 with warfarin (rate ratio 1.07, 95% CI (0.57–1.89)). Although this projected event rate was essentially equivalent between WATCHMAN and warfarin, the confidence interval exceeded the pre-specified non-inferiority margin of 1.75 and, therefore, failed to prove non-inferiority. The second co-primary efficacy endpoint (stroke or systemic embolism >7 days post-randomisation) was 0.025 vs. 0.020 (risk difference 0.0053 [95% CI –0.0190 to 0.0273]), achieving non-inferiority. Based upon the results of PROTECT AF and PREVAIL, the WATCHMAN device was approved by the FDA in March 2015 to reduce the risk of thromboembolism in patients with non-valvular AF who are not candidates for long-term anticoagulation therapy.

A patient-level meta-analysis of the WATCHMAN trials (PROTECT-AF and PREVAIL) and their continued access registries (Continued Access to PROTECT and Continued Access to PREVAIL [CAP2]) was recently published [21]. This meta-analysis included 2406 patients with 5391 patient years of follow-up. The rates of haemorrhagic stroke, non-procedural bleeding, and cardiovascular death were reduced in patients who received LAA closure compared with patients on long-term oral anticoagulation. However, once peri-procedural complications are included, all-cause stroke and systemic embolism were similar between the two groups, and there was no significant difference in all-cause mortality nor in major bleeding complications. A slightly increased risk of haemorrhagic stroke in the warfarin arm was offset by an increased risk of ischaemic stroke in the WATCHMAN group. The increased risk of ischaemic stroke persisted even after exclusion of strokes in the first 7 days. This finding suggests that warfarin continues to confer benefit over WATCHMAN in the long term for ischaemic stroke, probably because not all strokes in AF are due to emboli from the LAA.

The largest prospective patient cohort was collected by 47 centres between October 2013 and May 2015 and included 1021 patients who were scheduled for LAA occlusion with the WATCHMAN device in the EWOLUTION registry [22]. Implantation success was 98.5% with no or minimal residual flow achieved in 99.3% of implanted patients. The 7‑day procedural/device-related serious adverse event rate was 2.8% with 30-day mortality rate of 0.7%. The rates of procedural success and 7‑day serious adverse events compared favourably with those found in PROTECT-AF (88% and 7%, respectively) and PREVAIL (95.1% and 4.2%, respectively) trials. In particular, the rate of procedural/device-related stroke was 0.1% through 30 days, compared with rates of 0.9% in PROTECT AF and 0.4% in PREVAIL trials. Interestingly, 62% of patients were deemed to be ineligible for oral anticoagulation, a group that had not been included in the previous trials. Patients who were ineligible for oral anticoagulation had even lower 30-day serious adverse event rates compared with the group eligible for oral anticoagulation (6.5 vs. 10.2%, *p* = 0.042). Sixty percent of patients in the EWOLUTION registry were treated with dual-antiplatelet therapy during follow-up, with further results to be presented at upcoming meetings.

The global experience with percutaneous LAA closure predominantly involves patients who are ineligible for oral anticoagulation. To date, no randomised trials have been performed in this patient population. The largest registry experience with the WATCHMAN device in this patient cohort is the ASAP (ASA Plavix Feasibility Study with WATCHMAN Left Atrial Appendage Closure Technology) study which enrolled 150 patients with a contraindication to anticoagulation with both aspirin and clopidogrel for 6 months after device implantation instead of the standard warfarin [23]. During a mean duration of follow-up of 14.4 months, the primary efficacy outcome of all-cause stroke or systemic embolism occurred at a rate of 2.3% per year and ischaemic stroke occurred at a rate of 1.7% per year. This rate was lower than that in the PROTECT AF study (2.2% strokes per year) despite not using warfarin. After a median follow-up of 55.4 months (range 1.2 to 75.6 months), the annualised ischaemic stroke or systemic embolism was 1.8%, which did not appreciably differ to that reported at 14.4 months. This rate was lower than the annual 7.3% rate expected for this cohort if they were receiving aspirin alone [24]. These findings support the approach that WATCHMAN may be safely used without oral anticoagulation; however, further trials are still necessary to determine best practice.

### Amplatzer cardiac plug

The Amplatzer Cardiac Plug (ACP, St. Jude Medical, St. Paul, Minnesota, USA) received CE mark approval in Europe in 2008. The design features a distal lobe which anchors to the body of the appendage and a proximal disc which seals the ostium of the appendage. The distal lobe contains six pairs of barbs designed to increase stability within the appendage. The second generation ACP device (Amulet) received CE marking in 2013 and has replaced the ACP in Europe. The Amulet can accommodate larger LAAs (up to 32 mm) (Fig. 2c). Following its initial release, the Amulet was subsequently temporarily removed from the market to redesign the delivery system for ease of use.

In contrast with the WATCHMAN device, the only published reports of the ACP devices are retrospective, non-randomised case series. The lack of a control group in these studies precludes inferences about the comparability of these devices with contemporary treatment. The initial European experience was published in 2011 reporting the results of 143 patients who underwent LAA occlusion with the ACP device. Although this first registry showed the initial experience of several operators with the device, the percentage of procedural success reached 96% and the rate of complications was relatively low with no intra-procedural deaths [25].

The largest retrospective multicentre study to date included 1047 patients from 22 European and Canadian centres. Procedural success was 97.3%, with a periprocedural major adverse event rate of 5%. This included 8 procedure-related deaths, 9 strokes, 13 cases of cardiac tamponade and 13 major bleeds. The one year all-cause mortality was 4.2%, no deaths were device related. The annual rate of systemic thromboembolism and major bleeding was 2.3% (59% risk reduction) and 2.1% (61% risk reduction), respectively [26].

ACP implantation has been marketed for use with antiplatelet therapy only, despite little supporting evidence. Currently, the most followed antithrombotic regimen post-ACP implantation is DAPT for 3–6 months followed by a single antiplatelet agent. An ongoing clinical trial in Europe, ELIGIBLE (Efficacy of Left Atrial Appendage Closure After Gastrointestinal Bleeding) (NCT01628068), is designed to compare the ACP device with aspirin and clopidogrel versus oral anticoagulation alone.

### WaveCrest occluder device

The WaveCrest device (Coherex Medical Inc, Salt Lake City, Utah, USA) consists of a single-lobe, nitinol-based design for occluding the LAA. The device is covered by a foam layer on the LAA side to promote rapid endothelialisation and PTFE on the side facing the left atrium to reduce thrombus formation (Fig. 2d). The Wavecrest device differs from others in that the occluding atraumatic face of the device is deployed first into the LAA at the ostium and advanced outside the delivery sheath, without requiring delivery sheath placement into the LAA itself. This is of advantage if the LAA is too small to accommodate deeper devices such as the WATCHMAN or ACP. In addition, the occluder and anchoring system can be operated independently, allowing repositioning before anchoring. Once in position, the deployment anchors are advanced into the LAA body.

The Wavecrest I trial (multicentre, prospective, non-randomised registry) studied 73 patients with non-valvular AF who underwent LAA closure using the Wavecrest device. Initial results were presented at the Transcatheter Cardiovascular Therapeutics (TCT) and EuroPCR meetings showing a 93% acute procedural success with 3 mm or less peri-device flow at 6 weeks in 96% of patients. There were two pericardial effusions, but no procedural stroke, device embolisation, or device-associated thrombus were reported. The final results of this trial are still pending. The device was approved in Europe in 2013 with plans to conduct clinical trials leading to regulatory approval in the United States.

## Epicardial LAA closure device

### LARIAT

The LARIAT device (SentreHeart, Redwood City, California, USA) is a transcatheter LAA ligation system that utilises both the endocardial and epicardial approach to place a surgical knot around the ostium of the LAA and approximate all walls, thus excluding the LAA. The system consists of three components: 1) EndoCATH: a 15 mm compliant occlusion balloon catheter; 2) FindrWIRZ: 0.025 inch and 0.035 inch magnet-tipped guidewires and 3) LARIAT: a 12 F suture delivery device. The procedure involves pericardial and transseptal access, placement of the endocardial magnet-tipped guidewire in the apex of the LAA with balloon identification of the LAA ostium, followed by connection of the epicardial and endocardial guidewires forming a rail for the suture to be advanced and to capture the LAA. The LARIAT procedure may be considered when the LAA is too large for either the WATCHMAN or the ACP devices, as long as the maximal ostial diameter is less than 40 mm. Theoretically, anticoagulation is not required with the LARIAT system as no foreign body is left behind on the endocardial surface of the left atrium. The LARIAT system device is approved by the FDA for soft tissue closure (approximation) only, but not specifically for prevention of thromboembolism with LAA occlusion.

The safety and efficacy of the LARIAT procedure were reported in several observational studies. In a single-site study of 89 relatively low-risk patients with AF, the placement of the LARIAT device was successful in 96% and there were no associated complications [27]. Complete closure was confirmed in 98% of patients at 1 year. No late strokes thought to be embolic were documented. Despite the encouraging results of the study, it is important to note that 25% of initially screened patients were subsequently excluded due to LAA size or morphology, presence of LAA thrombus, or pericardial adhesions precluding pericardial access.

Subsequent multicentre results from the United States showed higher periprocedural complication rates. The retrospective series of 154 patients reported by the United States Transcatheter LAA Ligation Consortium showed a procedural success of 94% defined as a residual leak less than 5 mm. Major complications were observed in 9.7% with major bleeding (9.1%) and significant pericardial effusion (10.4%) being the most frequent [28]. In another United States multicentre series, among 41 consecutive LARIAT procedures, procedural success was 93%. However, pericardial effusion requiring drainage was 20%, perforation of the LAA occurred in 9%, and 17% had pericarditis [29].

In July of 2015, the FDA issued a safety communication stating that cases of death and complications such as perforation of the heart or complete LAA detachment from the heart associated with the use of LARIAT had been reported. These real-world data raised concerns about the procedural safety of this device. Patients being considered for this procedure in the United States are typically those with absolute contraindications to anticoagulation, or who have anatomies that are unsuitable for endovascular closure.

## Emerging technologies

Numerous next-generation LAA closure technologies are in various stages of development and clinical testing. Opportunities for enhanced outcomes include (1) suitability for more anatomies, (2) complete closure of the left atrium minimising peri-device leaks, (3) eliminating procedural complications and (4) improving long-term stroke reduction.

LAmbre (Lifetech Scientific Corp, Shenzhen, China) is a self-expanding nitinol-based device consisting of a hook-embedded umbrella with a cover that is connected to a short central waist [30]. The Cardia Ultrasept LAA Occluder (Cardia, Eagan, Minnesota, USA) consists of a distal cylindrical bulb anchoring into the LAA and a separate articulated sail unfolding the ostium. The Occlutech LAA Occluder (Occlutech International AB, Helsingborg, Sweden) is a conical-shaped self-expanding device that is anchored with closed loops at the distal device margin [31].

The LAmbre and Occlutech devices recently received CE mark approval for LAA closure.

## Future directions

Anticoagulation therapy has been demonstrated to be efficacious in decreasing stroke risk and remains the first option in eligible patients. In those patients in whom oral anticoagulation is contraindicated, LAA exclusion may be considered. Transcatheter LAA closure is a relatively new frontier that continues to evolve. Similar to other structural heart procedures, LAA device occlusion will benefit from a multidisciplinary team approach to maximise program success and should be restricted to a few expert centres so that complication rates are kept to a minimum.

As evidence accumulates regarding the benefit of LAA closure in reducing the incidence of stroke in patients with AF, several remaining issues still need to be addressed. No device thus far has been adequately studied in a randomised controlled fashion in the high-risk AF population that cannot receive anticoagulation or been directly compared with NOACs to determine best practice. PRAGUE-17 is an ongoing randomised controlled trial comparing transcatheter left atrial appendage closure and NOACs (NCT02426944).

Furthermore, continued prospective studies are still necessary to demonstrate each device’s long-term efficacy and safety as well as to determine specific anticoagulation or antiplatelet regimens that may be necessary. New techniques need to be developed to enhance device placement, such as better guiding catheters, safer ways to access the pericardial space, and enhanced imaging, in order to make these procedures safer and reduce the procedural time.

Another important aspect of left atrial appendage closure is cost and reimbursement. Despite the encouraging clinical data from many studies, reimbursement for LAA closure remains challenging in many countries. The cost effectiveness of LAA closure for stroke prevention has recently been demonstrated in comparison with pharmacological treatment based on trial data [32, 33]. It is important that payers recognise the potential clinical and economic benefits of these devices and, therefore, it is critical that cardiology professional societies continue to engage the payers to improve the current reimbursement status. Despite all these challenges, we are optimistic that this niche field will continue to evolve with new technologies potentially acting as game changers.
